# Socio-environmental exposures and health outcomes among persons with sickle cell disease

**DOI:** 10.1371/journal.pone.0175260

**Published:** 2017-04-06

**Authors:** Monika R. Asnani, Jennifer Knight Madden, Marvin Reid, Lisa-Gaye Greene, Parris Lyew-Ayee

**Affiliations:** 1 Sickle Cell Unit, Caribbean Institute for Health Institute, The University of the West Indies, Mona, Kingston 7, Jamaica (W.I.); 2 Tropical Metabolism Research Unit, Caribbean Institute for Health Institute, The University of the West Indies, Mona, Kingston 7, Jamaica (W.I.); 3 Mona GeoInformatics Institute, The University of the West Indies, Mona, Kingston 7, Jamaica (W.I.); UNITED STATES

## Abstract

There is much variability in the expression of sickle cell disease (SCD) and recent works suggest that environmental and social factors may also influence this variability. This paper aims to use geographic information systems technology to examine the association between socio-environmental exposures and health outcomes in all persons who have attended or currently attend the Sickle Cell Unit in Jamaica. Rural patients presented for clinical care at older ages and had less annual visits to clinic. Persons travelled relatively long distances to seek SCD care and those travelling longer had less health maintenance visits. Urban patients had a higher prevalence of significant pain crises (69.4% vs. 55.8%, p value<0.001) and respiratory events (21.2% vs. 14%, p value<0.001). Prevalence of leg ulcers did not vary between rural and urban patients but was higher in males than in females. Females also had lower odds of having respiratory events but there was no sex difference in history of painful crises. Persons with more severe genotypes lived in higher poverty and travelled longer for healthcare services. Persons in areas with higher annual rainfall, higher mean temperatures and living farther from factories had less painful crises and respiratory events. The paper highlights a need for better access to healthcare services for Jamaicans with SCD especially in rural areas of the island. It also reports interesting associations between environmental climatic exposures and health outcomes.

## Introduction

The term sickle cell disease (SCD) includes a variety of pathological conditions resulting from the inheritance of the sickle haemoglobin (HbS) gene either homozygously or as a compound heterozygote with other interacting abnormal haemoglobin genes. It is the most common monogenic hereditary disorder, affecting millions of persons worldwide and is especially prevalent in persons of African and Asian descent [1]. About 300,000 babies are born with the disease annually. Jamaica is a Caribbean country where the prevalence of an abnormal haemoglobin gene is ~15% and one out of 150 babies born has a sickle haemoglobinopathy [2].

The primary cause of the clinical symptomatology of sickle cell disease is the intracellular polymerization of sickle hemoglobin (HbS) that occurs when sickle erythrocytes are partially deoxygenated under the hypoxic conditions of the microcirculation [3]. The clinical manifestations of the disease include repeated painful vaso-occlusive, haemolytic, aplastic episodes, and sequestration crises. Complications may affect various organs and systems, mainly skeletal, genito-urinary, gastrointestinal, spleen, hepato-biliary, cardiopulmonary and central nervous system [4].

There is much variability in the expression of disease and many factors have been implicated, e.g. genetic factors. In more recent years, environmental and social factors are also emerging as possibly influencing this variability. Studies from U.K. have demonstrated increased hospital admissions in SCD to be significantly associated with increased wind speed and low humidity, but show no relationship to temperature, rainfall or barometric pressure [5], whereas several other studies have shown an increase in painful crises in cold and rainy seasons [6–8]. Air quality has been shown to have a significant effect on acute pain in SCD where high levels of ozone (and low levels of carbon monoxide and nitric oxide were associated with increased numbers of hospital admissions [9,10]. Inhaled nitric oxide has been observed as possibly beneficial for acute vaso-occlusive crisis in pediatric patients [11], while zinc supplementation has been associated with decreased incidence of infections, number of hospitalizations, and vaso-occlusive pain crisis [12].

Socioeconomic conditions and geographic location also play a role in the variation seen in SCD outcomes. Sex is found to be a determinant in many clinical outcomes in SCD where men have been found to have lower life expectancy [13,14], higher prevalence of pain crises[15] and lower nitric oxide bioavailability [16]. Males also tend to have poorer health seeking behaviours and later presentation for healthcare [17].

Other findings suggest that utilization of health care services is lower in patients living in rural areas, even though they have lower physical functioning and higher socioeconomic distress levels [18,19]. Our group in Jamaica has reported that rural patients assessed their quality of life to be higher than their urban counterparts, despite similar physical functioning scores [20]. We attributed these findings possibly to differences in social as well as other, e.g. environmental, factors.

Geographic Informational Systems (GIS) methods provide a set of tools for describing and understanding the changing spatial organization of health care, for examining its relationship to health outcomes and access, and for exploring how the delivery of health care can be improved [21]. It can enhance the understanding of the association between contaminants in the environment and disease [22]. GIS technology has been useful in analyzing the patterns of disease spread during the 2003 severe acute respiratory syndrome (SARS) outbreak in Hong Kong [23], and more recently the deadly Ebola virus [24].

The Sickle Cell Unit (SCU) in Jamaica has one of the largest clinic populations of persons with SCD in the world and has been operating over a period greater than 40 years. There is no similar facility available throughout the rest of the island. In this project, we aim to utilize GIS technology to determine the environmental and social exposures of persons with SCD attending SCU and seek further understanding of their relationships, if any, to health outcomes in SCD. Specifically, we hypothesize that patient factors such as sex and genotype, and geo-social exposures such as rural/urban residence, poverty, mean temperature, rainfall, and elevation above sea level will have an association with common clinical presentations in SCD such as pain crises, respiratory problems and leg ulcers. Additionally, we wish to examine whether health care utilization, specifically annual attendance for routine, health maintenance care or attendance for acute care, is associated with patient factors, such as sex and genotype and other geo-social variables, exposures such as distance patients travel to seek health care at the SCU, at what age patients first registered at the SCU, and how long they have been attending the SCU.

## Materials and methods

This was a population based, observational study requiring a database link of all patients of the SCU to available information housed at Mona GeoInformatics Institute (MGI). The study was granted ethical approval by The University of the West Indies Ethics Committee. All persons who had been registered at the SCU up to December 2011 were geocoded based on their residential addresses.

The Unit is located in an urban setting and provides free healthcare to patients with SCD across island. It operates a daily clinic at its main site located in the eastern region and two monthly outreach clinics in the southern and western regions of the island. There are about 6,000 active patients registered in its database and the Unit has close to 10,000 annual visits. Healthcare is provided by primary care physicians, paediatricians, nurses, and a social worker. Medical technologists are available to provide urgent hematology assessments daily. Chronic blood transfusion programs are not employed by the Unit. Hydroxyurea use is increasing but uptake is still quite low. Patients presenting with acute complications are admitted to the daycare ward located within the SCU, and patients requiring hospitalization for inpatient care are transported to the nearest government operated hospitals.

Once geocoded, spatial data layers attached to community and/or enumeration district data were created for further processing and analysis with physical, environmental and social data, all housed at MGI. MGI has access to 30-year meteorological records such as rainfall, mean temperature and surface elevation from the Meteorological Office of Jamaica, from their more than 300 monitoring stations island-wide, and to geochemical data (from the International Centre for Environmental and Nuclear Sciences’ Geochemical Atlas of Jamaica, 1995 [25]) across more than 250 monitoring sites across the island with details on available soil elements including zinc, lead, cadmium, and arsenic. Distances of patients from factories and SCU clinics were calculated, poverty levels (based on Planning Institute of Jamaica’s database), and rural/urban designations by communities were also used for the analyses. The poverty indicators utilized were those that best predicted per capita consumption levels in households based on data from the Planning Institute of Jamaica. Poverty level was defined as being the percentage of persons in that community living below the poverty line.

Clinical data extracted from the SCU database on these individuals included their date of birth, gender, genotype, age at first visit to clinic, number of years attending the SCU, total number and annual rate of all visits to clinic, total number and annual rate of health maintenance visits, as well as history of and annual rates of visits for specific conditions, such as pain events, leg ulcers and respiratory problems including acute chest syndrome and asthma. Genotype was further categorized as mild (including heterozygous SC and Sβ+ thalassemia genotypes) and severe (including the homozygous SS and the heterozygous Sβ0 thalassemia genotypes) genotypes.

### Statistical analysis

All analyses were performed using the ESRI™ suite of ArcGIS 10.3 software and Stata/SE Software version 12.1 for Windows™.

Descriptive statistics were reported as frequency and percent for categorical variables and as mean and standard deviation or medians and interquartile range (IQR) as appropriate for continuous variables. Thereafter, social, environmental and clinical variables were compared by sex, by mild/severe genotype, and by rural/urban residence of participants. Mean, medians and frequencies were compared using two-sample t-test, the Wilcoxon rank sum test, and the chi-squared test as applicable.

Multivariable regression analyses were conducted to determine social, including sex and poverty, and environmental predictors of health care utilization, namely annual rate of all visits to the SCU and the annual rate of health maintenance visits to the SCU. Logistic regression analyses were conducted to study predictors of common clinical outcomes, such as painful crises, respiratory events and leg ulcers.

## Results

### Socio-demographic characteristics of the study population

Table 1 reports on baseline characteristics of the clinic sample. A total of 8504 persons [4,129 (48.6%) males, 4,373 (51.4%) females] had been registered to the SCU to study end date. Of these, 2,407 (28.3%) resided in rural communities and 6,097 (71.7%) in urban centres. About 43.6% of patients were registered at the SCU before their 5^th^ birthday and another 35.8% registered between 5 and 18 years of age.

**Table 1:** Profile of study population by urban/rural residence.

Variable	Urban	Rural	Total	p-value
	(n = 6,097; 71.7%)	(n = 2407; 28.3%)	(n = 8,503)	
**Gender, M (%)**	2945 (48.3)	1184 (49.2)	4129 (48.6)	0.46
**Genotype: Severe (SS and Sβ0 thalassemia) (%)**	4035 (66.8)	1752 (73.5)	5787 (68.7)	***<0*.*001***
**Poverty index (median,IQR)**	2, 13	24, 18	8, 20	***<0*.*001***
**Dependency Ratio (mean±SD)**	0.60±0.17	0.76±0.14		***<0*.*001***
**Ratio of SCD vs. general population, % (mean±SD)**	0.54±0.32	0.39±0.29		***<0*.*001***
**Distance from clinic, kilometers (mean±SD)**	16.4±14.8	32.3±16.2		***<0*.*001***
**Age at first visit, %**				***<0*.*001***
**0-<5 years**	45.1	39.7	43.5	
**5-<18 years**	34.4	39.4	35.8	
**18-<30 years**	13.2	13.8	13.4	
**>30 years**	7.3	7.1	7.3	
**Annual health maintenance visits (median,IQR)**	0.79, 1.1	0.85, 1.1	0.81, 1.1	0.96
**Annual SCU visits (median,IQR)**	2.6, 2.7	2,2, 2.1	2.5, 2.5	***<0*.*001***
**Total years at SCU, (median,IQR)**	11.2, 16.9	8.1, 13.3	10.2, 16.1	***<0*.*001***
**History of Pain experience, % Yes**	69.4	55.8	65.6	***<0*.*001***
**Annual Pain events rate,(mean ±SD)**	0.42, 0.75(n = 4234)	0.34, 0.58(n = 1344)	0.40, 0.70	***0*.*06***
**History of respiratory events, % Yes**	21.2	14.0	19.1	***<0*.*001***
**Annual respiratory illness rate (median,IQR)**	0.17, 0.36(n = 1290)	0.16, 0.37(n = 336)	0.16, 0.36	0.13
**History of leg ulcers, % Yes**	22.0	21.0	21.8	0.33
**Annual leg ulcer rate, (mean ±SD)**	0.29, 0.71(n = 1343)	0.30, 0.77(n = 506)	0.29, 0.73	0.054
**Mean temperature,** ^**o**^**C(mean±SD)**	30.2±0.9	29.6±1.0	30.1±1.03	***<0*.*001***
**Average elevation, kilometers (mean±SD)**	0.04, 0.08	0.18, 0.31	0.06, 0.14	***<0*.*001***
**Average rainfall, meters (median,IQR**	1.1, 0.45	1.76, 0.57	1.25, 0.7	***<0*.*001***
**Distance from factories, kilometers (median,IQR)**	0.80, 1.12	4.3, 4.8	1.2, 4.8	***<0*.*001***

**Fig 1:**
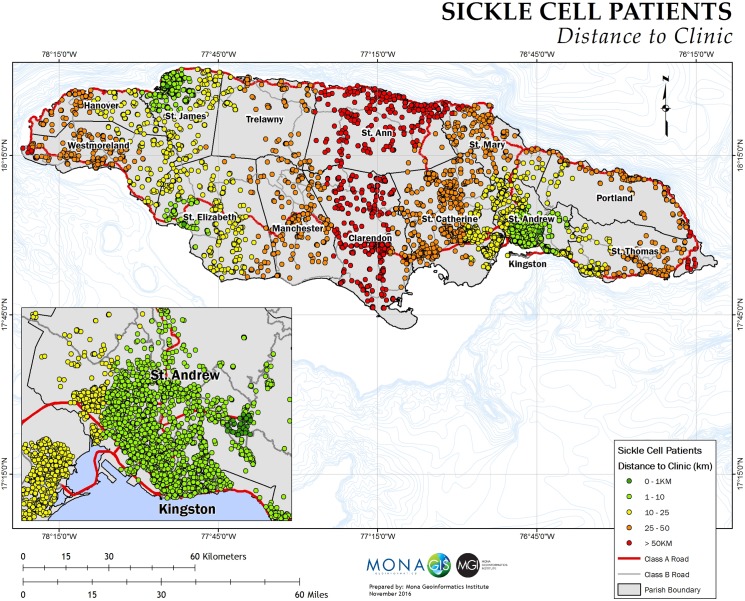
Distance from a sickle cell clinic in kilometres.

The mean ratio of SCD patients to general population by communities was 0.5% ± 0.3% and ranged between 0.02% and 4.8%. The mean poverty index was 12.0% ± 13.3%. Patients travelled a mean distance of 20.9±16.8 kilometers to access SCU services (Fig 1). The total number of visits made to the SCU by patients ranged between 1 to 1,096 visits (mean total visits: 40.0±53.0 visits) over a time span of 6 months to 60 years (mean time attending SCU: 12.9±11.2 years).

### Sex differences in social and clinical characteristics

There were no gender differences in levels of poverty, genotype, annual health maintenance visits, or exposure to physical conditions such as rainfall, temperature, elevation above sea level or distance from factories. However, males lived further from a sickle cell clinic (mean distance: 21.3 vs. 20.5 kms; p value: 0.035), had higher annual visits to the clinic (mean visits: 3.2 vs. 3.0; p value: 0.008), and had higher prevalence of respiratory events (52.5% in males vs. 47.5% in females; p value<0.001) and of leg ulcerations (55.6% in males vs. 44.4% in females; p value<0.001). There was no gender difference in prevalence of pain events.

### Social & clinical characteristics by genotype severity

Over two-thirds (68.7%) of patients had the more severe genotypes (Hb SS and Sβ0 thalassemia), 65.6% had suffered from significant pain crises, 21.7% had suffered from leg ulcers, and 19.1% had experienced some respiratory complication such as acute chest syndrome or asthma. Those with severe genotypes had significantly higher experiences of pain crises (68.4% vs. 60.0%; p vale <0.001); leg ulcers (28.8% vs. 6.6%; p value<0.001) and respiratory events (21.8% vs. 13.6%; p value<0.001). Persons with more severe genotypes when compared to mild genotypes were more likely to be diagnosed at a younger age (Less than 5 years of age: 47.1% vs. 35.5% respectively; 5–18 years of age: 35.5% vs. 36.9% respectively; p value<0.001), and had a higher rate of annual visits (3.4±2.8 vs. 2.5±1.7 visits; p value <0.001) as well as health maintenance visits (1.1±0.98 vs. 0.96±0.89, p value<0.001) to the SCU. Those with more severe genotypes had higher poverty index than those with the mild genotypes (12.7±13.5% respectively vs. 10.4±12.6%, respectively; p value<0.001) and lived farther distances from SCU services (21.5±16.9 kms. vs. 19.6±16.3 kms respectively; p value<0.001).

### Rural/urban disparities

Table 1 also reports significant findings between rural and urban patients. 73.5% of rural patients versus 66.8% of urban patients had the more severe genotypes (p value <0.001) and rural patients had to travel significantly longer distances to access SCU services. Poverty levels and correspondingly dependency ratio were higher for rural patients. Ratio of SCD persons to general population was significantly higher in urban areas. More urban patients were registered to the SCU before the age of 5 years. There were no differences in the annual health maintenance visits for the two groups, but rural patients had lower rates of total annual visits to SCU as well as lower total number of years attending the SCU.

**Fig 2:**
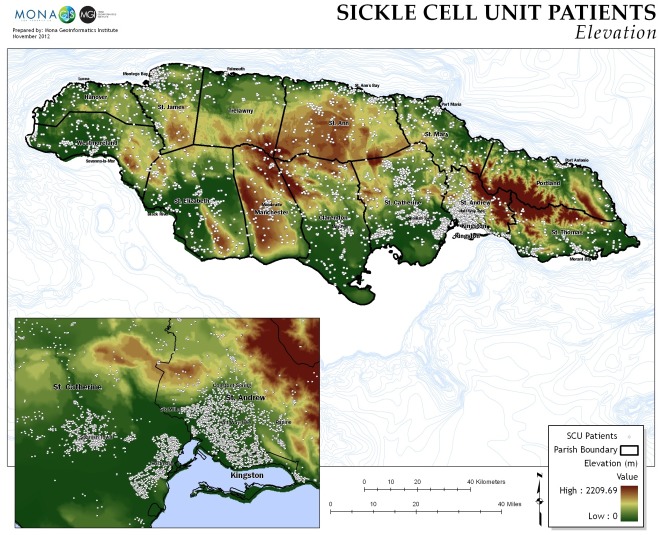
Elevation above sea level in metres.

**Fig 3:**
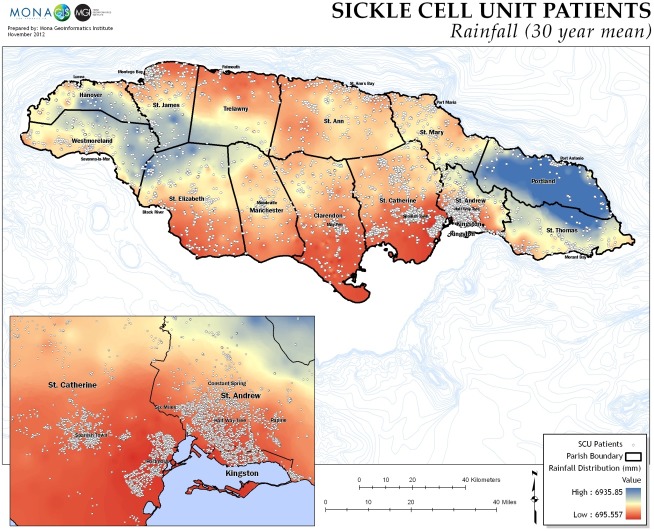
Rainfall in millimetres, mean of 30 years.

Rural patients lived further from factories and had higher mean elevation above sea-level (Fig 2), higher annual rainfalls (Fig 3), and lower mean temperatures than their urban counterparts.

Annual visits for leg ulcers were somewhat higher in those from rural residences, whereas there were no differences in annual visits for pain events or respiratory problems. Urban patients had a higher prevalence of significant pain crises (69.4% vs. 55.8%, p value<0.001) and respiratory events (21.2% vs. 14%, p value<0.001). Prevalence of leg ulcers did not vary between rural and urban patients.

### Predictors of health services utilization

Table 2 reports on variables that predict rates of annual total visits and annual health maintenance visits to the SCU. Persons living in higher poverty and those in rural areas had lower rates of annual total visits to the clinic. Persons with more severe genotypes had higher rates of annual total visits as well as annual health maintenance visits. Rural patients did not differ from their urban counterparts in rates of annual health maintenance visits, but those who had to travel longer distances had lower rates of annual health maintenance visits. Those who joined the clinic at an older age and those who had been attending the clinic for a longer time had lower rates of annual health maintenance visits as well. There was no effect of sex on rates of visits in both regression models.

**Table 2:** Multivariate regression models examining predictors of health services utilization in Jamaicans with SCD.

**Outcome**	**Predictors**	**Coefficient (95% C.I.)**
**Annual total visits to SCU**	Poverty	-0.025 (-0.03 to-0.02) **
	Rural residence^1^	-0.32 (-0.46 to -0.17) **
	Severe genotype^2^	1.02 (0.90 to1.14) **
	Females^3^	-0.07 (-0.18 to 0.04)
	Years attending SCU	-0.047 (-0.05to -0.04) **
	Age at 1^st^ visit to SCU	-0.048(-0.05 to -0.04) **
	Distance from clinic	-0.004 (-0.008to -0.001)
**Model Adj R^2^: 0.13; p value <0.001**		
**Annual health maintenance visits**	Poverty	-0.004 (-0.006 to -0.002) **
	Rural residence^1^	-0.021 (-0.07 to 0.03)
	Severe genotype^2^	0.19 (0.15 to0.23) **
	Females^3^	0.04 (-0.001 to 0.07)
	Years attending SCU	-0.038 (-0.039 to -0.036) **
	Age at 1^st^ visit to SCU	-0.03 (-0.03 to-0.028) **
	Distance from clinic	-0.002 (-0.003 to -0.0002)*
**Model Adj R^2^: 0.30; p value <0.001**		

*p<0.05

**p<0.001

^1^Reference category is ‘Urban residence’

^2^Reference category is ‘Mild genotype’

^3^Reference category is ‘Males’

### Predictors of common clinical outcomes

Table 3 reports on some social and environmental predictors of three common clinical outcomes in SCD, namely painful crises, leg ulcers and respiratory problems such as acute chest syndrome and asthma. Adjusting for poverty in all models showed no effects on all 3 clinical outcomes.

**Table 3:** Logistic regression examining predictors of common clinical outcomes in Jamaicans with SCD.

**Outcome**	**p-value**	**Predictors**	**Odds Ratio (95% C.I.)**
**History of painful crises**	<0.001	Rural residence^1^	0.70 (0.62 to 0.80) ***
		Severe genotype^2^	1.57 (1.42 to 1.73) ***
		Females^3^	1.00 (0.92 to1.10)
		Annual rainfall	0.63 (0.57 to 0.71) ***
		Elevation above ground level	0.75 (0.49 to 1.15)
		Distance from factories	0.98 (0.96 to 0.99)**
		Mean temperature	0.83 (0.77 to 0.89) ***
**History of respiratory problems**	<0.001	Rural residence^1^	0.84 (0.71 to 0.99)*
		Severe genotype^2^	1.90 (1.67 to 2.16) ***
		Females^3^	0.81 (0.73 to0.90) ***
		Annual rainfall	0.60 (0.51 to 0.70) ***
		Elevation above ground level	0.70 (0.41 to 1.22)
		Distance from factories	0.97 (0.94 to 0.99)**
		Mean temperature	0.86 (0.79 to 0.95)**
**History of leg ulcers**	<0.001	Rural residence^1^	0.95 (0.82 to1.11)
		Severe genotype^2^	5.99 (5.01 to 7.08) ***
		Females^3^	0.67 (0.60 to 0.75) ***
		Annual rainfall	0.80 (0.70 to 0.92) ***
		Elevation above ground level	0.99 (0.60 to 1.63)
		Distance from factories	0.99 (0.97 to 1.01)
		Mean temperature	0.90 (0.82 to 0.98)*

*p<0.05

**p<0.01

***p<0.001

^1^Reference category is ‘Urban residence’

^2^Reference category is ‘Mild genotype’

^3^Reference category is ‘Males’

Persons living in rural areas, those living in areas with higher annual rainfall, those living in areas with higher temperatures, and those further from factories had lower painful crises and respiratory problems. Persons with the more severe genotypes had greater odds of having these clinical complications.

Rural/urban residence was not predictive of leg ulcers, whereas those with more severe genotypes had greater odds of having had leg ulcers. Persons living in areas with higher annual rainfall and those living in areas with higher temperatures had lower odds of having had leg ulcers.

After adjustment for other social and environmental variables, females had 19% lower odds of history of respiratory events and 33% lower odds of having a history of leg ulcerations, but there was no sex difference in history of pain events.

## Discussion

Wide variability exists in expression of SCD between individuals, and at least some of this variability is attributable to social factors including the environment. Whereas the burden of SCD lies in low and low-middle income countries with tropical climates, most studies examining environmental influences have been conducted in higher income countries, which also happen to have more temperate climates [26]. This paper examines in great detail many of these socio-environmental influences on health outcomes in SCD. Jamaica has one of the oldest comprehensive care centres for SCD, and as it is a tiny island, many persons living in the island may opt to seek care at the SCU facility. The maps presented also highlight this point as there is a cross-island distribution of persons registered at the SCU. It is significant that rural persons’ access to clinic for health maintenance did not differ from urban persons as they apparently see value in these visits. Rural persons had higher prevalence of the more severe genotypes and might be possibly due to the fact that ones with milder genotypes do not make the effort to attend for healthcare. Additionally, the rural clinics are held only once monthly (this is a limitation of this study as the rural sample might be underrepresented), and so it is understandable why their annual rate of total attendance to the SCU is lower than that of their urban counterparts. Rural persons especially have to travel large distances to seek care and they continue to present later for care. Hence there is a need for more facilities to be developed where SCD specific care will be available,and greater access to SCD care more proximally may decrease that gap and possibly improve outcomes.

Rural persons also had higher poverty levels, tended to be diagnosed later and lived further from clinic. Health care is currently a free commodity in the island but all services are not always available in the public sector. These disparities highlight further the need for facilitating health care utilization by creating better access to care, as those who are in higher poverty had lower health maintenance visits.

There were not many sex disparities in the Jamaican SCD population. Even though Jamaican males are generally known to have poorer health seeking behaviours [17] associated with worse health outcomes, the SCD males had similar rates of annual health maintenance visits but higher rates of annual total visits when compared to SCD females in this study. Males had higher prevalence of leg ulcers (similar to what exists in previous literature [27,28]) and respiratory events. They also tended to have their first episode of significant pain crisis, respiratory event and leg ulcerations at lower ages than girls did. As most of these complications of SCD began in early adolescence, a hormonal role may be implicated in explaining this sex difference in clinical outcomes.Further studies are needed to continue to explore these differential outcomes between males and females.

There are interesting weather related relationships discerned in this paper. Higher annual rainfall and higher temperatures were associated with lower prevalence of all three clinical outcomes examined. Persons who lived further distances from factories also had lower prevalence of painful and respiratory events. Further research would be needed to dissect the possible implications of this finding. The effects of higher altitude (as assessed by elevation above sea level) disappeared once temperature was added to the models. It has been widely reported that exposure to cold in persons with SCD results in higher pain experiences [7,8,10,26] and our results corroborate these findings even within a narrow range of tropical temperatures between 25°C to 30°C. Environmental pollutants are increasingly being understood as being causative in disease, especially respiratory and allergic conditions [29,30]. There may be direct effect of particle components on generating added reactive oxygen species and further driving oxidative stress and inflammatory responses [31]. As these latter mechanisms are already implicated as being patho-biological in SCD [32], then the effect of environmental pollutants in SCD is probably multiplicative. These findings are critical in ongoing education among patients and families and advising preventative measures, especially in avoiding cooler temperatures and living close to factories and exposure to other means of air pollution. The effect of higher rainfall being consistently and independently protective against these clinical outcomes seems counterintuitive but will need greater study. Studies in the U.K. [33] and France [10] have found no influence of rainfall on outcomes whereas previous study in Jamaica has shown a positive association of rainfall with painful events [7].

In conclusion, the paper highlights that the disease is prevalent island wide and the distance persons are currently travelling to access healthcare services is long. The latter is further associated with poorer health seeking behaviours, especially for health maintenance. Persons are still presenting relatively late for healthcare and early childhood care is crucial to better health outcomes and in reducing childhood mortality associated with SCD [34]. Newborn screening will alleviate this problem but will need to be supported by more widely available and accessible health care. The latter will also reduce health disparities that rural persons and persons living in higher poverty face.

The paper also contributes significantly by highlighting important, independent effects of several social (especially rural/urban residence) and environmental conditions on clinical outcomes in a large SCD population. Environmental conditions deserve further study to understand their underlying mechanisms and possible interactions so that appropriate interventions can be recommended for persons living with this very serious disorder.
